# Quantitative Trait Locus Analysis

**Published:** 2000

**Authors:** Judith E. Grisel

**Affiliations:** Judith E. Grisel, Ph.D., is an assistant professor in the Department of Psychology, Furman University, Greenville, South Carolina

**Keywords:** quantitative trait locus, genetic mapping, genetic polymorphism, DNA, animal strains, animal model, genotype, phenotype

## Abstract

Alcoholism is a quantitative disorder that is caused by the combined influences of numerous genes (i.e., quantitative trait loci [QTLs]) and environmental factors. To identify QTLs for alcoholism, researchers compare subject groups (e.g., inbred mouse strains) that differ in both their genetic makeup (i.e., genotype) and alcohol-related trait (e.g., sensitivity to certain alcohol effects). Using statistical tests one can then determine whether a specific gene or DNA region contributes to the trait of interest. This strategy requires that the relevant gene exists in several variants (i.e., is polymorphic). To conduct such QTL analyses, researchers study either a large population of mice that all differ in their genotypes or compare several strains, each of which has a fixed genotype. However, QTL analyses still have several limitations. Nevertheless, such studies already have identified several DNA regions and genes that may affect the response to alcohol and thus may contribute to the risk for alcoholism.

Alcohol’s effects are dependent upon both genetic and environmental factors as well as the interaction of these broad sources of influence (e.g., Shuckit 1998). In order to better understand, treat, and prevent problems associated with alcoholism, research has focused on pinpointing the specific genetic and environmental elements that contribute to a liability for, or protect against, alcohol abuse. Adding to the complicated nature of the disease, all cases of alcoholism are not caused by the same set of factors.

As with other complex traits, alcoholism is a quantitative or polygenic disorder in which the influences of multiple genes combine to contribute to the pathological state (e.g., Enoch and Goldman 2000). Unlike qualitative (i.e., Mendelian) traits, which have an “either-or” expression pattern in a population and are generally mediated by a single gene, quantitative traits, such as height or intelligence, vary continuously across a population and derive from a constellation of both genetic and environmental influences. Virtually all behavioral characteristics (i.e., phenotypes) are quantitative traits. The genes (or DNA regions that contain the genes) influencing a quantitative trait are called quantitative trait loci (QTLs). This article describes some considerations for the genetic analysis of quantitative traits, the general steps involved in QTL analysis, the primary strengths and failings of such approaches, and a few examples of how QTL analysis has helped researchers identify genes contributing to differential sensitivity to alcohol.

## Strategies for Analyzing Quantitative Traits

In the absence of evidence implicating a specific gene (i.e., a candidate gene) that may contribute to a quantitative trait, researchers can employ several strategies to determine the role of an individual DNA sequence among all the other genetic and nongenetic influences on the trait. For instance, investigators can analyze genetic differences between a human population with a specific trait or prone to that trait (e.g., alcoholics) and a control population (e.g., nonalcoholics), using either linkage or association studies.^1^ Such analyses could allow researchers to identify genes whose structure or expression differs between the two populations. The ultimate success of such an endeavor, however, depends largely on a clear prognosis of a person’s risk for the disease and on a set of distinct causes (i.e., etiology). For alcoholism, this process is made more difficult by the fact that the disease derives from a complex interplay of many genetic and environmental influences that are not uniform across alcoholics.

Studies in laboratory animals, most commonly in rodents, provide some distinct advantages over human studies in the quest for elucidating the genetic causes of a quantitative trait, such as alcoholism, even in the absence of specific hypotheses regarding its etiology. First, one can isolate more easily the genetic and environmental influences in laboratory animals than in humans, because to a large extent, one can control the animals’ experimental environment. Consequently, one can attribute virtually all the variance in phenotype across different animal strains to genetic factors.

Second, many animal strains used in these studies already have been well characterized with respect to their genetic makeup (i.e., genotype). For instance, a list of strain-specific polymorphic markers and genes—that is, DNA regions that exist in different forms within a test population—is readily accessible from database sources.^2^ Thus, two strains that differ both in behavior and genotype may provide a good starting point for genetic analysis.

Third, one can design animal models to isolate a specific aspect of the alcoholic phenotype (e.g., alcohol preference, tolerance, or dependence) in order to clarify this aspect’s specific etiology. As a result, researchers can parcel the heterogeneous disorder into units that are more amenable to genetic analysis. Thus, the mechanisms underlying alcohol preference, tolerance, or dependence can be studied independently. Factors identified (e.g., genes) as contributing to one of these phenotypes then can be evaluated for a more general role in other aspects of alcoholism.

Finally, a high degree of genetic conservation exists between the human genome and the genome of commonly used animal models, such as mice, indicating that large portions of the chromosomes have remained relatively intact across species during evolution. In fact, researchers have estimated that approximately 80 percent of the mouse genome is organized similarly to the human genome. This finding makes it likely that the location of a mouse DNA segment that influences a trait of interest suggests the location of a corresponding gene in the human genome (Copeland et al. 1993).

## Mapping QTLs

In general, QTLs are identified by correlating genetic variation with trait variation; a significant correlation between genotype and phenotype suggests that DNA status helps determine trait expression. QTL mapping can be conducted by comparing various inbred strains (i.e., strains in which each animal has the same genotype) or by comparing animals from a population with diverse genotypes (i.e., a genetically segregating population). Following the initial screen in which a DNA region has been identified that contributes to a specific phenotype, subsequent testing can be used to further resolve the exact location and mechanism of the gene effect.

To identify QTLs, researchers evaluate groups of subjects that have polymorphic genes (i.e., genes that exist in more than one form) for a differential sensitivity to a particular alcohol effect (e.g., sedation). They conduct statistical tests to determine whether specific DNA regions contribute to the variance in alcohol sensitivity. If the response depends on the sequence of a certain DNA region, then the region is a QTL and contains either a gene that influences the quantitative trait or a DNA sequence that modifies the expression of such a gene.

It is crucial for such analyses that the test subjects are polymorphic for the relevant genes; one cannot identify QTLs in groups that do not have genetic differences in the critical regions, even if genes in those regions may be influencing the response. As a general rule, the greater the number of polymorphic DNA markers, the greater is the statistical likelihood of finding significant correlations. However, this strategy has practical limitations, the primary one being that an estimated 100,000 genes in the mouse are contained within approximately 3 billion DNA building blocks (i.e., base pairs) (O’Brien et al. 1999). In spite of the fact that great progress has been made in unraveling the mouse genome, researchers are still a long way from a physical map of the DNA that would make gene identification truly facile.

### Strategies for QTL Mapping

Mice are the species of choice for most QTL mapping studies, because thousands of polymorphic markers are currently known in this species, many of which have been genotyped across strains (Belknap et al. 1997). Researchers generally use one of two mapping strategies: (1) an analysis of a large population of mice with different genotypes or (2) the testing of several strains that each have a fixed genotype.

**Figure 1:**
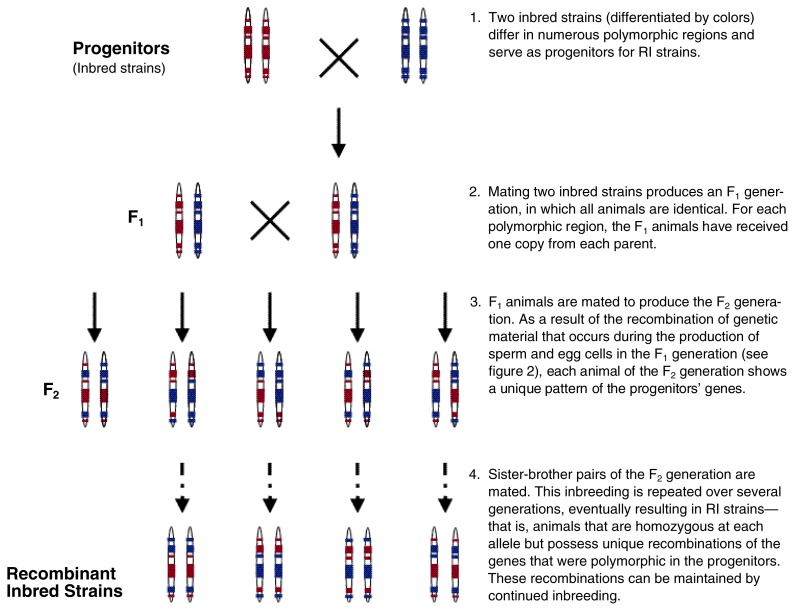
Generation of recombinant inbred (RI) strains. The progenitors of RI strains are animals of two inbred strains that carry different forms (i.e., alleles) of polymorphic genes (indicated by the colors blue and red). For each animal, the figure shows one chromosome pair in which one chromosome is inherited from each parent. In an inbred strain, each animal has two copies of the same allele of each gene, meaning they are homozygous at each locus. Many alleles do not differ between strains, however, and the white areas on the chromosomes represent those regions that are not polymorphic.

**Figure 2:**
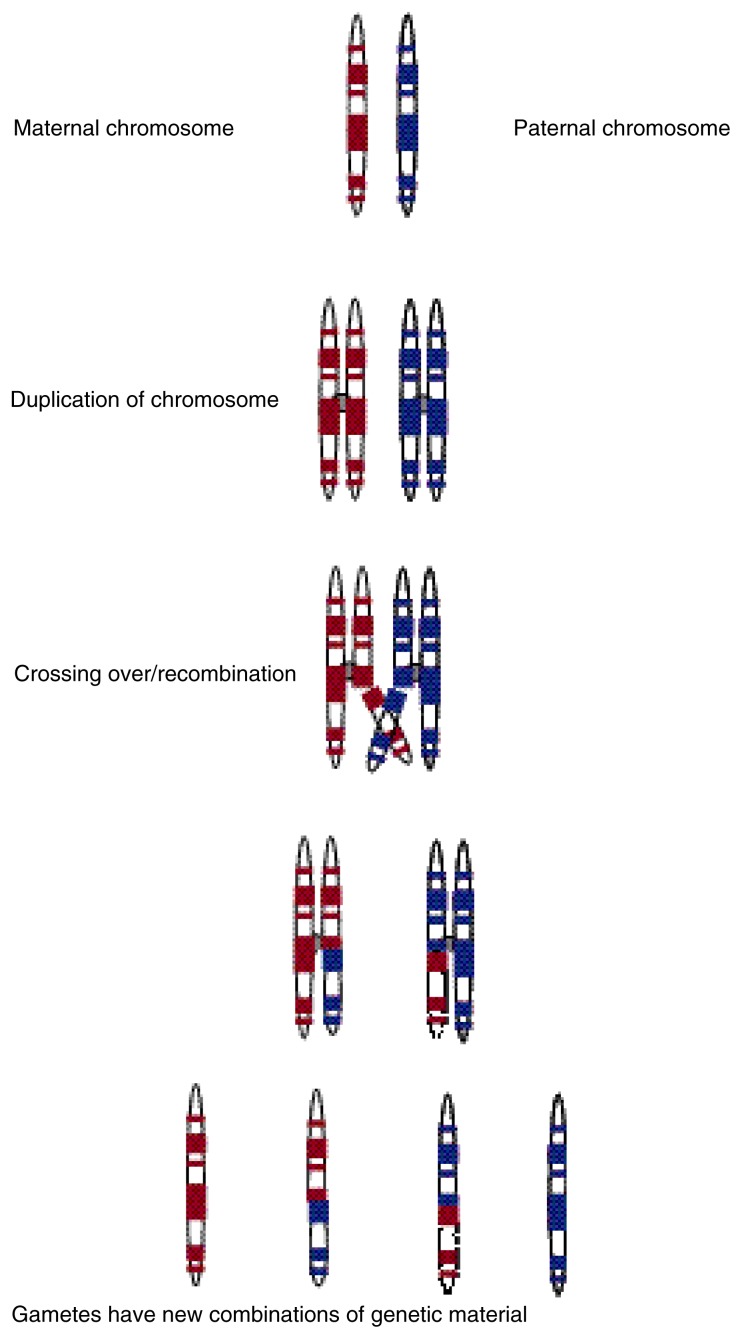
Crossing over and genetic recombination during the specialized cell division (i.e., meiosis) that results in the production of sperm or egg cells (i.e., the gametes). Each member of each chromosome pair (i.e., the one inherited from the mother and the one inherited from the father) duplicates itself, and genetic material may cross over. In inbred strains, in which each chromosome pair is identical, crossing over has no observable effect. In animals in which the maternal and paternal chromosomes are not identical (e.g., F_1_ animals), however, crossing over will result in the recombination of parental genes. Because of this recombination, all the genes on a chromosomes are not always inherited together.

For the first strategy, so-called F_2_ mice are a popular choice. These mice are derived from the cross of two inbred strains (see figure 1). In inbred strains, all animals have the identical genotype, and each animal has two identical copies of each gene^3^—that is, the animals are homozygous at each locus. Strains used in gene mapping often have known genetic diversity or large phenotypic differences for a trait of interest. The offspring of a cross of two such strains (called F_1_ animals) will have two different alleles of each gene that differs between the strains. The F_1_ animals all are identical, however, because at each locus where the parent strains differed, each F_1_ animal has received one allele from each parent. Mating animals from the F_1_ generation produces the F_2_ generation. In contrast to the F_1_ animals, each F_2_ animal possesses a unique combination of progenitor alleles. This diversity results from the recombination of DNA segments between the two members of a chromosome pair that occurs during the specialized cell division process (i.e., meiosis) in the production of egg and sperm cells and which results in a recombination of the progenitor DNA (see figure 2 for an illustration of gene recombination during meiosis). Thus, the F_2_ animals are a genetically diverse group of subjects.

When analyzing such a group, researchers determine the test score for the trait for each animal and then attempt to correlate the individual test scores with the polymorphic markers. For example, investigators might test a large group of F_2_ animals for their voluntary alcohol consumption and then look for systematic genetic differences between the highest and lowest consuming animals. Although limiting the analysis to the extremes of the trait of interest may decrease its ability to detect QTLs with only small effects, it will markedly reduce the costs associated with analyzing the animals’ genotypes. Overall, the greatest advantage of analyzing F_2_ animals is that given a sufficiently large population (i.e., sufficiently large number of genotypes), one can detect and map QTLs with relatively small effects in a single study.

The second major strategy for mapping QTLs—called a two-step or multistep approach—requires sequential testing of more than one population. Most often, this strategy involves a set of recombinant inbred (RI) strains. RI strains are generated by mating two inbred strains and then inbreeding the F_2_ generation of this cross. This strategy results in the recombination of the alleles present in the progenitors and in the stable transmission of the recombined alleles in a set of related RI strains that are bred to homozygosity (see figure 1). The advantage of this strategy is that many RI strains whose genotypes have been well characterized are commercially available.

Two sets of RI strains have been frequently employed in alcohol research. The BXD RIs, which were derived from C57BL/6J and DBA/2J progenitors, comprise 26 strains with more than 1,300 genotyped polymorphic alleles distributed across the genome. The LSXSS RIs, which were derived from mice selected for high and low sensitivity to alcohol’s sleep-inducing (i.e., hypnotic) effects, include 27 strains with more than 180 known polymorphisms. This latter set has been particularly useful for identifying QTLs underlying the sensitivity to the hypnotic effects of depressant drugs, including alcohol.

**Figure 3:**
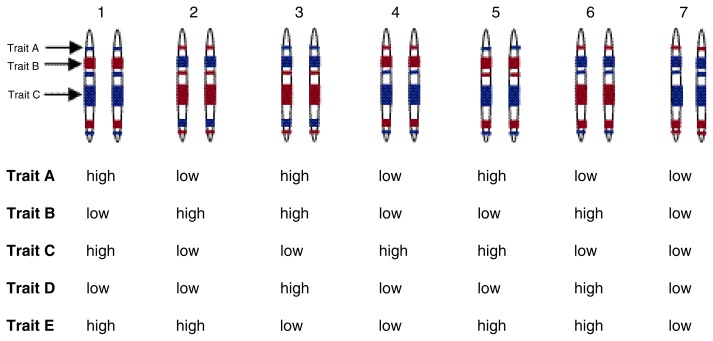
Quantitative trait loci (QTL) analysis of recombinant inbred strains. The example shows seven different strains. For each strain, a single chromosome pair is depicted that is associated with three traits (i.e., A, B, and C). Chromosome regions inherited from each progenitor (differentiated by color) confer either high or low responses for the corresponding trait. High correlations between the trait and the genetic status indicate a QTL. For example, for trait A, the gene form (i.e., allele) inherited from the blue progenitor confers a high response, whereas the allele inherited from the red progenitor confers a low response. Conversely, for traits B and C, the red alleles confer a high response and the blue alleles confer a low response. More numerous recombinations increase the ability to resolve regions of influence. Two other traits (i.e., D and E), however, are not associated with QTLs on this chromosome, because no correlation exists between the levels of those traits and any of the chromosome regions analyzed.

With either the two-step or multistep mapping approach, researchers test mice from these or other inbred strains for the phenotype of interest (e.g., alcohol consumption level). The mean values obtained for each strain are then correlated with the strains’ genotypes (see figure 3). Because the number of “subjects” to be included in such an analysis is really the number of available genotypes or strains, much fewer genotypes exist than with the F_2_ approach. As a result, this approach has less statistical power than the F_2_ approach and, consequently, a relatively high likelihood of missing some significant associations (i.e., type II errors, which are described in the following paragraph).

### Limitations of QTL Mapping

QTL mapping has been likened to casting a wide-mesh net across the entire sea of genetic information. As such, this method is a powerful tool for conducting initial screens of the whole genome; however, this method is also subject to some statistical and practical pitfalls. For example, a single experiment can detect only QTLs with a relatively large influence on the trait of interest. Most QTLs, however, probably have only a small influence, and thus researchers may miss some relevant loci using this technique. Such errors are called “false negatives” or type II errors. Furthermore, because researchers analyze a large number of markers for their linkage with the trait, some correlations will appear positive purely by chance and thus are called “false positives” or type I errors.

Type I errors can be minimized by establishing stringent criteria for what is considered a significant correlation. The more stringent these criteria are, however, the more likely it is that some QTLs with small influences will be missed, increasing the chance of type II errors. To achieve a balance between these two concerns, the scientific community has relaxed the initial criterion for significant correlations in QTL screening while insisting on confirmation of potential QTLs in followup experiments.^4^

Another limitation of QTL mapping is that when researchers locate a QTL using one of the strategies described earlier, it does not mean that they have found a gene contributing to the trait of interest. The DNA regions identified as QTLs are still so large that they likely contain hundreds of genes. Several methods exist, however, that can help narrow the DNA region enough to conduct a practical search for a specific gene (for an overview of some of these techniques, see Darvasi 1998).

One such strategy uses congenic strains—mice in which a particular region of DNA (e.g., a QTL) from one strain is transferred (i.e., introgressed) into the genome of another strain, followed again by inbreeding (for a more detailed description of this process, see Crabbe et al. 1999). Using this strategy, one can produce several congenic strains that carry different or overlapping parts of the original QTL. By identifying strains with similar phenotypes and determining which QTL regions they contain, one can discriminate DNA regions most likely to contain the relevant gene(s). The greater the number of such congenic strains (and thus of specific QTL regions), the finer or more precise the mapping will be.

## QTL Mapping in Alcohol Research

Despite its limitations, QTL mapping is a powerful method for widespread analyses of the genetic influences underlying quantitative traits, such as those contributing to alcoholism. Researchers have provisionally identified numerous QTLs as affectors of alcohol responses; of those QTLs, approximately 15 to 20 have resulted in confirmed loci (for a recent review, see Crabbe et al. 1999). These loci influence such phenotypes as alcohol self-administration, alcohol-induced sedation, and withdrawal. For example, the LSXSS RI strains were used to screen for QTLs influencing sedation induced by alcohol and other depressant drugs. Followup analyses in over 1,000 F_2_ mice revealed four significant QTLs contributing to this phenotype.

In some cases, the identification of a QTL will readily suggest a candidate gene. For example, using a two-step strategy with the BXD RIs, researchers identified three loci that accounted for nearly 70 percent of the gene-mediated differences in acute alcohol withdrawal (Buck et al. 1997). One of these QTLs mapped to a region of mouse chromosome 11 that contains the genes for several components of a specific receptor for the brain chemical (i.e., neurotransmitter) gamma-aminobutyric acid (GABA). Alcohol is known to influence neurotransmitter activity at this GABA_A_ receptor; furthermore, GABA mediates many effects of sedative-hypnotic drugs such as alcohol. Consequently, the genes encoding the GABA_A_ receptor components appeared to be good candidate genes for contributing to alcohol withdrawal. Further investigation supported this hypothesis, suggesting that a polymorphism involving a single building block (i.e., amino acid) in one of the components of the GABA_A_ receptor complex may be responsible for differences in alcohol withdrawal severity (Buck and Hood 1998). Whether this polymorphism also plays a role in human alcohol withdrawal or in the vulnerability to alcoholism remains to be seen. Nonetheless, the value of such an approach leading to a putative mechanism for alcohol sensitivity and to a potential difference in risk for alcoholism is obvious.

## Conclusions

QTL mapping in animals provides a valuable, though preliminary, contribution to the search for genes contributing to human alcoholism. The identification of QTLs—even though these regions typically contain about 15 million DNA base pairs and an estimated 500 to 1,000 genes—provides a significant advance in the daunting task of elucidating the genetic bases of alcohol effects. The subsequent identification of novel candidate genes and the clarification of their mechanisms of influence contribute to the eventual understanding of functional variability in humans that confers differential vulnerability to alcoholism.

## Footnotes

1 For a definition of this and other technical terms used throughout this issue, please see the central glossary, pp. 193–195.

2 For example, the Jackson Laboratory maintains a list of rodent markers at its Web site <www.informatics.jax.org>.

3 Many genes exist in different variants, or alleles. Each organism carries two copies of each gene, one inherited from the mother and one inherited from the father. If both gene copies carry the same allele, the organism is said to be homozygous for that gene. If the two gene copies carry different alleles, the organism is said to be heterozygous for that gene.

4 Lander and Kruglyak (1995) have proposed a widely accepted standard for a significance criterion using logarithm-of-the-odds-of-linkage (LOD) scores.
